# Short-Term Influence of Surya Namaskar on Trunk Musculature Flexibility in School-Going Children

**DOI:** 10.7759/cureus.96780

**Published:** 2025-11-13

**Authors:** Dhwani D Chanpura, Pinal Ghelani

**Affiliations:** 1 Pediatric Physiotherapy, College of Physiotherapy, Sumandeep Vidyapeeth Deemed to be University, Vadodara, IND; 2 Physiotherapy, College of Physiotherapy, Sumandeep Vidyapeeth Deemed to be University, Vadodara, IND

**Keywords:** effect of surya namaskar, flexibility, physical activity, school-aged children, trunk muscles flexibility, yoga

## Abstract

Background: Yoga, a holistic approach to enhancing physical and mental health, includes physical postures, controlled breathing, and meditation, promoting a balance between body and mind. Surya Namaskar, a classical yogic sequence, is known for its benefits such as improved flexibility, strength, and mental focus. For school-going children, it is thought that regular practice of Surya Namaskar helps in improving posture, which is essential for long hours of sitting during school activities, improves attention, and reduces stress, which can help them to manage with improved concentration and develop better self-awareness. Over time, Surya Namaskar's practice has been found to support injury prevention, muscle relaxation, and overall wellness.

Methods and procedures: A total of 100 students in the sixth and eighth grades participated in this study. The modified-modified Schober test was used to assess trunk muscle flexibility before and after Surya Namaskar practice. The children were instructed to do a warm-up, followed by 10 slow Surya Namaskars with a 10-second hold for each asana, and finished with a cool-down session.

Result: Out of the 100 participants, 36 (36%) were male and 64 (64%) were female. Thirty-three (33%) students were from the sixth grade, 35 (35%) from the seventh grade, and 32 (32%) from the eighth grade. The results from both tests showed improvement in post-values when compared to pre-values, demonstrating a positive effect of the intervention. Additionally, pre-post comparisons showed statistical significance for gender, standard, and BMI, with p < 0.05.

Conclusion: The findings of this study suggest that practicing Surya Namaskar has significant positive effects on promoting better posture and alignment of the whole body, improving flexibility in lumbar muscles, and preventing musculoskeletal injuries. Including Surya Namaskar in children’s daily routines can help maintain their overall physical and mental well-being.

## Introduction

Yoga, one of the world’s oldest practices, finds its earliest mention in the *Vedas* (around 1500 BCE). Initially rooted in spirituality, yoga aimed to promote meditation, prayer, and higher consciousness. Over centuries, its purpose expanded beyond spiritual enlightenment to include physical health, mental clarity, and stress regulation [1]. Today, yoga is widely integrated into healthcare and rehabilitation, recognized for its benefits in improving flexibility, posture, breathing, and overall well-being [2].

Among its many practices, Surya Namaskar (Sun Salutation) holds a special place as a complete workout that blends 12 asanas with controlled breathing [3]. This sequence not only enhances strength and flexibility but also stimulates internal organs, improves posture, and promotes spinal health. When practiced slowly, it tones muscles and aids relaxation, while faster rounds serve as an aerobic workout, boosting circulation and stamina. Regular practice has been shown to reduce muscle tightness, support joint mobility, balance energy systems, and improve physical as well as psychological health [4].

Flexibility, defined as the ability of joints and muscles to move freely through their full range, is essential for efficient movement and injury prevention. Limited flexibility often leads to musculoskeletal pain, poor posture, and reduced functional capacity in daily activities [5,6]. Previous studies highlight the long-term benefits of Surya Namaskar on flexibility, but there is limited evidence on its short-term effects, particularly when performed at a slower pace. 

With this gap in mind, the present study aimed to explore the short-term effects of slower-paced Surya Namaskar on trunk muscle flexibility in school-going children. The study aimed to assess the immediate effect of performing 10 slow-paced rounds of Surya Namaskar on trunk muscle flexibility in school-going children, using the modified-modified Schober test (MMST) as the measurable outcome. The intervention was designed to be feasible and practical within the school setting, making it attainable and relevant for promoting healthy posture and musculoskeletal well-being. Flexibility was evaluated before and after the same session, making the objective clearly time-bound.

## Materials and methods

This cross-sectional interventional study was conducted in in three private schools located in Vadodara District, Gujarat, India. The study was approved by the Sumandeep Vidyapeeth Institutional Ethics Committee (approval number: SVIEC/ON/PHYSIO/BNPG22/NOV/23/41) and registered in Clinical Trials Registry - India (CTRI) (registration number: CTRI/2024/01/061560). The intervention was low-risk, and participants were monitored throughout for any adverse effects. Written informed consent was obtained from the parents/guardians, and assent was obtained from the participating students.

Study population

Inclusion criteria were: children aged 11-14 years enrolled in sixth to eighth grades, able to follow simple verbal instructions, and with parental/guardian written consent and child assent. Exclusion criteria were: acute illness on the day of testing, known neurological or musculoskeletal disorders affecting spinal mobility, cardiovascular disease contraindicating exercise, current participation in structured sports or regular yoga training, and, for female student, being on their menstrual period on the day of testing.

**Figure 1:**
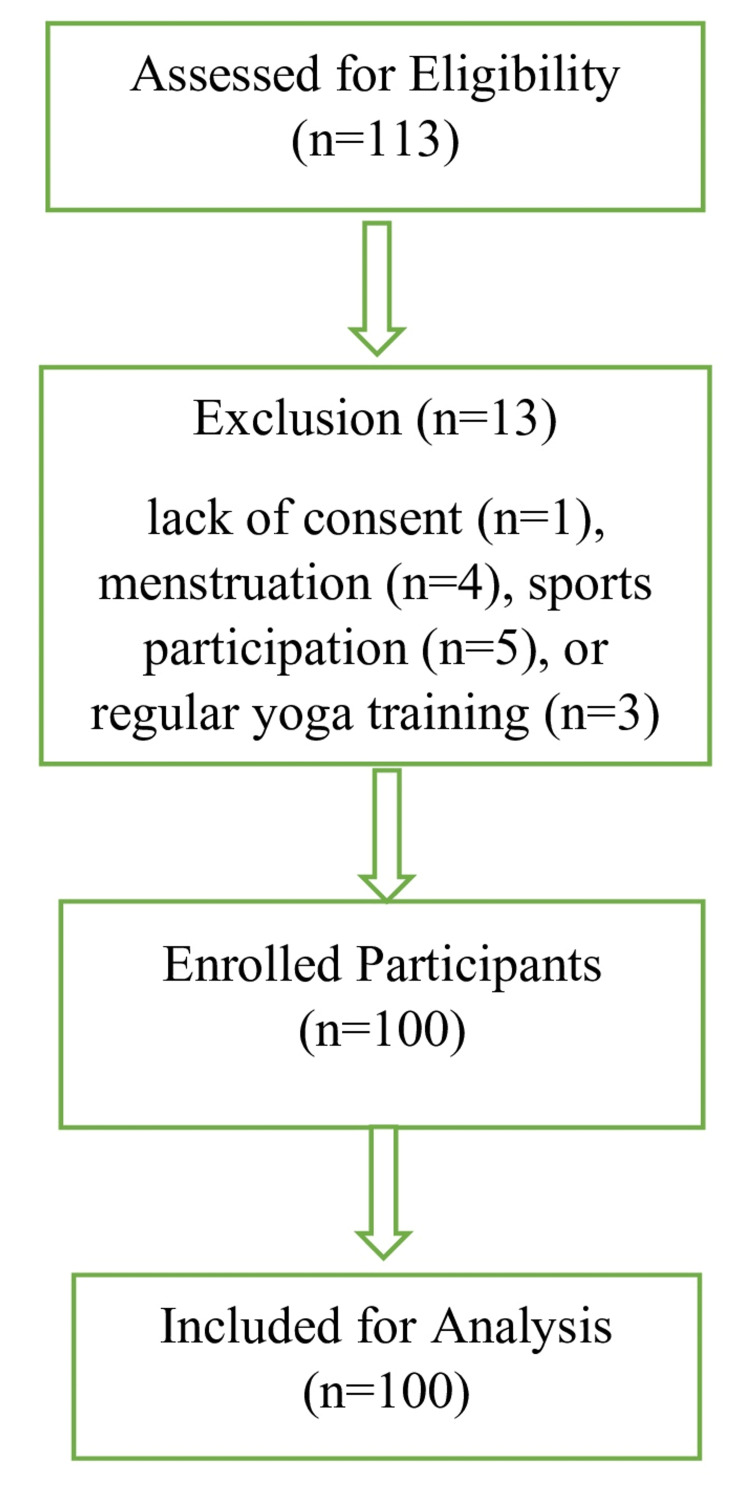
Flow diagram of participant recruitment

A total of 113 students from three private schools in Vadodara District were screened, of which 13 were excluded (due to lack of consent, menstruation, sports participation, or regular yoga training). The final study sample comprised 100 participants who met the eligibility criteria. The flow-chart for participant selection is given in Figure 1.

Intervention

Familiarization and Standardization

The Surya Namaskar postures are: (i) Pranamasana, (ii) Hasta Uttanasana, (iii) Pad Hastasana, (iv) Ashwa Sanchalanasana, (v) Parvatasana, (vi) Ashtanga Namaskara, (vii) Bhujangasana, (viii) Parvatasana, (ix) Ashwa Sanchalanasana, (x) Pad Hastasana, (xi) Hasta Uttanasana, and (xii) Pranamasana. All participants attended a brief familiarization demonstration where a certified yoga instructor and the study physiotherapist demonstrated each Surya Namaskar posture and the forward-bend technique used for the MMST. Participants practiced the sequence once under supervision to ensure correct form. All measurements were performed by a trained assessor who did not lead the Surya Namaskar instruction; the assessor remained blinded to subgroup hypotheses.

Pre-Test Procedures and Participant Instructions

Participants were asked to wear comfortable, non-restrictive clothing and to avoid vigorous physical activity for 24 hours before testing. On the test day, they were instructed to: (i) avoid heavy meals within one hour before testing, (ii) report any pain or discomfort, and (iii) perform usual daily activities prior to arrival. Before the pre-test measurement, participants completed a two-minute standardized warm-up consisting of light joint mobilization and walking in place.

Surya Namaskar (Standardized Procedure)

The intervention consisted of 10 slow-paced rounds of Surya Namaskar, each round comprising the classical 12-pose sequence. Each asana was performed deliberately with a 10-second hold at the end of the posture. The tempo and breathing pattern were standardized as follows: inhale on upward or opening movements (e.g., Hasta Uttanasana), exhale on forward or contracting movements (e.g., Pad Hastasana), and maintain slow, diaphragmatic breathing throughout. Transitions between asanas were slow and controlled; the certified instructor provided count-out guidance (seconds) so that each posture hold approximated 10 seconds. A single session, therefore, took approximately 20-25 minutes, including warm-up and cool-down. The instructor supervised all participants, corrected form as needed, and ensured knees were extended for the forward-bend portions used for MMST measurement.

Cool-Down

After completion of 10 rounds, participants performed a two-minute cool-down consisting of gentle self-stretching and breathing exercises.

Measurement and data collection

Participant demographic data (age, sex, height, weight, BMI, and school standard) were recorded.

MMST Detailed Measurement Protocol

**Figure 2:**
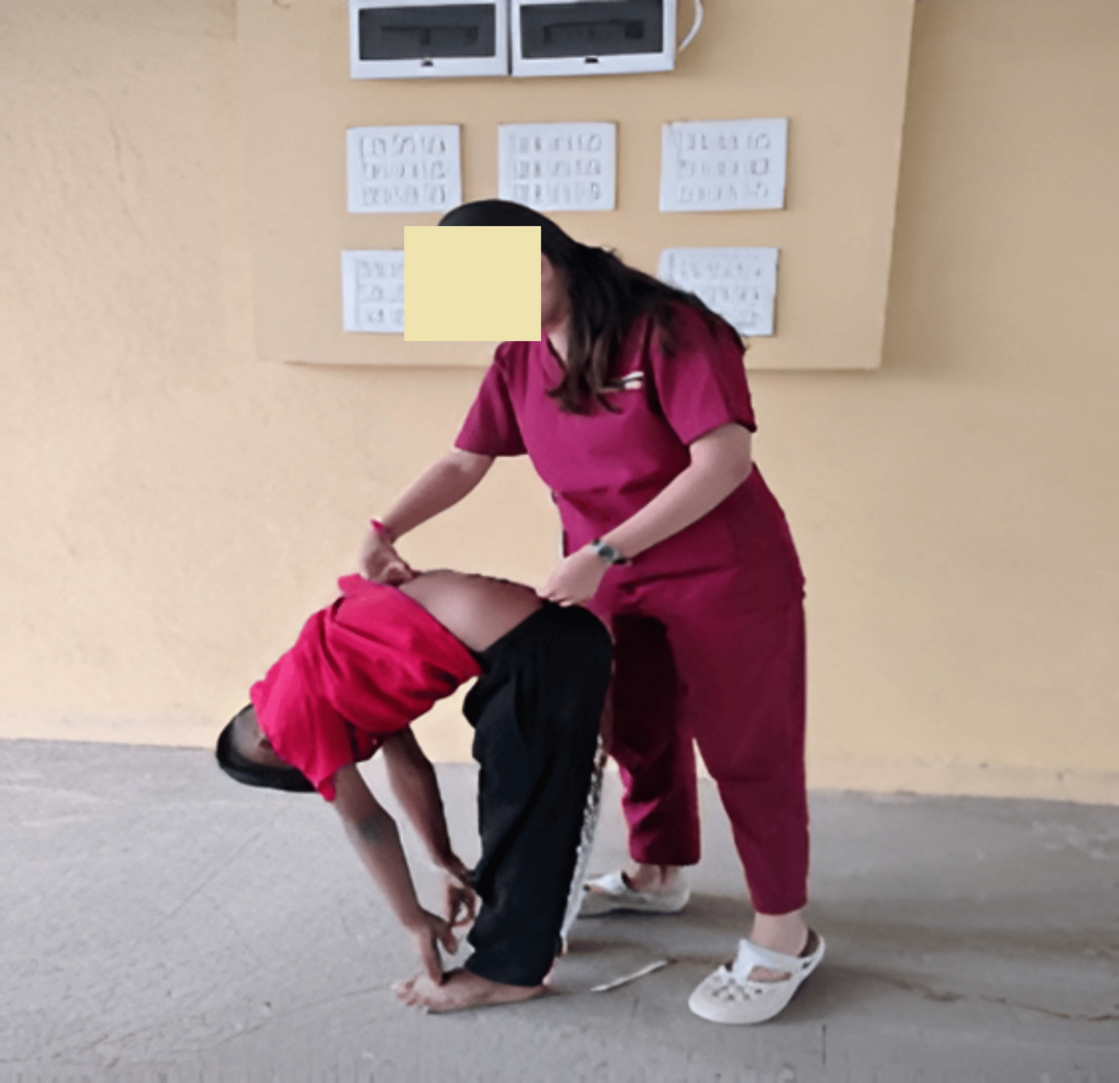
Modified-modified Schober test (MMST)

Trunk flexion (lumbar flexion) was measured using the MMST following a standardized procedure. The posterior superior iliac spines (PSIS) were palpated to identify the approximate level of the second sacral vertebra (S2). A skin-safe marker was used to mark the S2 level, and a second mark was placed 15.0 cm cranial to that point on the midline. A flexible tape measure (nearest 0.1 cm) was used to record the distance between the two marks in the upright standing position (baseline). The participant was then instructed to bend forward slowly with knees fully extended and hands sliding down the anterior legs as far as possible (without bending the knees and avoiding hip flexion compensations), and the new inter-mark distance was recorded at maximal comfortable flexion. The increase in distance (cm) between the two marks from standing to maximal forward bend represented the MMST value. For reliability, three consecutive trials were performed with a 15-30 second rest between trials; the mean of the three trials was used as the pre-intervention MMST value. The same procedure and the same assessor were used to collect the post-intervention measurements within two to five minutes after the cool-down. Care was taken to relocate the original skin marks for the post-measurement (or to re-mark at the same anatomical points if smudged), and the tape measure was applied with consistent tension (Figure 2) [7].

Measurement Quality Control

The assessor practiced the MMST protocol on pilot participants prior to data collection to ensure consistency. All measurement equipment (tape measure, marker) was the same for all participants. Where skin marks were unclear, the PSIS landmarks were re-palpated to ensure correct placement. The assessor recorded any deviations or participant discomfort in the study log.

Statistical analysis

Pre- and post-intervention MMST values were compared using nonparametric tests (Wilcoxon signed-rank test), and subgroup comparisons by gender, standard, and BMI category were conducted as specified in the secondary objectives. A flow diagram of recruitment and exclusion is provided.

## Results

A total of 100 students were included in the study. The distribution of participants across different grades was relatively equal, with 33 (33%) from the sixth grade, 35 (35%) from the seventh grade, and 32 (32%) from the eighth grade. The mean age of the participants was 12.02 ± 6.99 years. The average height and weight were 141.12 ± 7.50 cm and 34.45 ± 6.96 kg, respectively, with a mean BMI of 17.31 ± 2.83. Flexibility assessment was performed using hip flexibility and the MMST for trunk flexibility, and complete data from all 100 participants were included for analysis. 

The Wilcoxon test revealed significant improvements in post-intervention values compared to pre-intervention, with p < 0.05 indicating a positive effect of the intervention (Table 1).

**Table 1:** Pre and post intervention scores of the modified-modified Schober test (N=100)

Test	Mean±SD	Standard error	Median	Z value	P value
Pre	5.37±0.79	0.07	5.4	-8.741	<0.00001
Post	6.17±0.95	0.09	6.5		

Both male and female participants showed clear improvements from pre- to post-intervention, with statistically significant changes (p < 0.00001) (Table 2). This indicates that the intervention was highly effective in enhancing flexibility across sexes.

**Table 2:** The correlation of sex with pre- and post-values for the modified-modified Schober test (N=100)

Gender	Frequency (Percentage)	Test	Mean±SD	Standard error	Median	Z value	P value
Males	36 (36%)	Pre	5.39±0.83	0.13	5.4	-5.242	<0.00001*
		Post	6.25±0.93	0.15	6.5		
Females	64 (64%)	Pre	5.36±0.78	0.09	5.4	-7.022	<0.00001*
		Post	6.12±0.96	0.11	6.5		

Children from all three grades (sixth, seventh, and eighth) showed significant post-intervention improvements (p < 0.00001). The seventh-grade students demonstrated the greatest gain, followed by the eighth and sixth grades, indicating consistent effectiveness of the intervention across grades (Table 3).

**Table 3:** Correlation of academic grade (year) with pre and post values of the modified-modified Schober test (N=100)

Grade	Frequency (Percentage)	Test	Mean±SD	Standard error	Median	Z value	P value
6th	33 (33%)	Pre	5.21±0.76	0.13	5.2	-5.027	<0.00001*
		Post	5.8±0.88	0.15	5.8		
7th	35 (35%)	Pre	5.65±0.74	0.12	5.45	-5.301	<0.00001*
		Post	6.7±0.76	0.12	6.6		
8th	32 (32%)	Pre	5.21±0.82	0.14	5	-4.941	<0.00001*
		Post	5.95±0.95	0.16	5.8		

As shown in Table 4, both BMI groups (<18.5 and >18.5) showed significant improvements after the intervention (p < 0.00001). While gains were observed in both, participants with BMI >18.5 demonstrated slightly higher post-intervention flexibility scores.

**Table 4:** Correlation of BMI groups with pre- and post-values of the modified-modified Schober test (N=100)

BMI Group	Frequency (Percentage)	Test	Mean±SD	Standard error	Median	Z value	P value
<18.5 kg/m^2^	72 (72%)	Pre	5.33±0.84	0.1	5.25	-7.176	<0.00001
		Post	6.11±0.97	0.11	6.35		
>18.5 kg/m^2^	28 (28%)	Pre	5.45±0.7	0.12	5.4	-5.037	<0.00001
		Post	6.28±0.9	0.15	6.5		

## Discussion

This study explored the short-term effects of slow Surya Namaskar on trunk muscle flexibility in 100 school-going children. After a brief warm-up, participants performed 10 slow Surya Namaskars, holding each asana for 10 seconds, followed by cool-down exercises. Flexibility was assessed pre- and post-intervention using the MMST. Results showed significant improvements in MMST values from 5.37±0.79 cm to 6.17±0.95 cm (z = -8.337, -8.741; p < 0.00001).

The posture-enhancing effects of Surya Namaskar largely come from its cycle of repetitive stretching and strengthening. The 12 asanas involve holding, stretching, and relaxing, which gradually lengthen and smoothen muscles and connective tissues, allowing freer and more efficient movement. Strengthening elements further support mobility by ensuring stability during motion. On a structural level, changes in muscle fibers, such as sarcomere lengthening, and the natural viscoelastic properties of muscle (stress relaxation and creep) explain the immediate gains in flexibility often seen after static stretching [8,9].

The findings suggest that practicing Surya Namaskar can help prevent musculoskeletal issues linked to limited hip and lower back mobility, as these muscle groups, along with core strength and endurance, are essential for maintaining a healthy spine [10].

Our findings align with a study conducted on 50 female participants who performed 10 slow Surya Namaskars with 10-second holds. Flexibility of the hip and trunk was assessed using the modified Schober test, popliteal angle, and sit-and-reach test, and results confirmed immediate improvements supporting the outcomes of this study [4]. Similar findings were reported in a study on 15 girls aged 16-18 years. Participants were divided into two experimental groups performing Surya Namaskar at different paces (two and four minutes) and a control group, over six weeks. Flexibility assessed by the sit-and-reach test showed significant improvements in lumbar and hip mobility, highlighting the positive role of Surya Namaskar practice [11].

One study reported that improvements in the dynamic range of motion also enhance metabolic processes, as elevated muscle temperature reduces viscosity and allows smoother contractions, thereby increasing flexibility. Another study found that six weeks of practice led to significant gains in free movement [1,12].

Our findings are consistent with studies showing significant gains in flexibility after Surya Namaskar practice. One study of 30 female participants reported pre- and post-values of 4.67±1.50 and 5.73±1.53 following a six-week training protocol [13]. Other studies also confirmed Surya Namaskar as an effective method for enhancing joint flexibility and demonstrated similar improvements in middle elementary school students after six weeks of practice [3,14,15].

Evidence also supports the role of Surya Namaskar in improving both flexibility and agility. One study showed that dynamic Surya Namaskar significantly enhanced flexibility in physical education students [16]. Similarly, research on college male students reported a mean pre-post difference confirming notable improvements in flexibility as well as abdominal muscle strength with regular practice [13,17].

A study on 30 female college students found that Surya Namaskar significantly improved back flexibility and lumbar flexion [18]. Overall, evidence suggests that regular practice not only enhances joint mobility and physical function but also promotes pain-free movement and mental well-being, making it a valuable daily routine, particularly for children.

This study demonstrates the immediate benefits of Surya Namaskar on trunk flexibility using a standardized, supervised protocol in a school-based population, assessed with a reliable clinical measure (MMST). However, the findings are limited to short-term effects, with no follow-up to determine whether improvements are sustained over time. The study sample was restricted to a specific age group from one geographical region, which may limit generalizability. Flexibility was assessed using only a single outcome measure, and individual variations in posture performance could have influenced results. Future research should include multiple outcome measures, diverse study populations, and longitudinal follow-up to evaluate long-term effectiveness.

## Conclusions

The present study demonstrated that a single session of slow-paced Surya Namaskar led to an immediate improvement in trunk flexion, as measured by the MMST, indicating that it may be a simple and feasible short-duration activity to enhance spinal flexibility in school-going children. However, these findings reflect only short-term effects, and no conclusions can be drawn regarding long-term postural, musculoskeletal, or broader health outcomes.

While Surya Namaskar is generally considered safe and accessible, further studies with control groups, additional outcome measures, and longitudinal follow-up are needed to determine whether these immediate flexibility gains translate into sustained functional or postural benefits and potential roles in overall health promotion.
